# [Retracted] Salidroside mitigates hypoxia/reoxygenation injury by alleviating endoplasmic reticulum stress‑induced apoptosis in H9c2 cardiomyocytes

**DOI:** 10.3892/mmr.2024.13320

**Published:** 2024-09-02

**Authors:** Meng-Yao Sun, Da-Shi Ma, Song Zhao, Lei Wang, Chun-Ye Ma, Yang Bai

Mol Med Rep 18: 3760–3768, 2018; DOI: 10.3892/mmr.2018.9403

Following the publication of this paper, it was drawn to the Editor's attention by a concerned reader that certain of the cell apoptotic assay data shown in Fig. 1D on p. 3763 were strikingly similar to data that had already been submitted for publication in Fig. 3A in different form in another article written by different authors at different research institutes.

Owing to the fact that the contentious data in the above article had already been submitted for publication prior to its submission to *Molecular Medicine Reports*, the Editor has decided that this paper should be retracted from the Journal. The authors were asked for an explanation to account for these concerns, but the Editorial Office did not receive a reply. The Editor apologizes to the readership for any inconvenience caused.

